# Working From Home and Job Loss Due to the COVID-19 Pandemic Are Associated With Greater Time in Sedentary Behaviors

**DOI:** 10.3389/fpubh.2020.597619

**Published:** 2020-11-05

**Authors:** Cillian P. McDowell, Matthew P. Herring, Jeni Lansing, Cassandra Brower, Jacob D. Meyer

**Affiliations:** ^1^The Irish Longitudinal Study of Ageing, Trinity College Dublin, The University of Dublin, Dublin, Ireland; ^2^School of Medicine, Trinity College Dublin, The University of Dublin, Dublin, Ireland; ^3^Physical Activity for Health Research Cluster, Health Research Institute, University of Limerick, Limerick, Ireland; ^4^Department of Physical Education and Sport Sciences, University of Limerick, Limerick, Ireland; ^5^Department of Kinesiology, Iowa State University, Ames, IA, United States

**Keywords:** COVID-19, employment, work from home, physical activity, sedentary behavior

## Abstract

**Objectives:** Due to the COVID-19 pandemic, major changes to how, or even whether, we work have occurred. This study examines associations of changing COVID-19-related employment conditions with physical activity and sedentary behavior.

**Methods:** Data from 2,303 US adults in employment prior to COVID-19 were collected April 3rd−7th, 2020. Participants reported whether their employment remained unchanged, they were working from home (WFH) when they had not been before, or they lost their job due to the pandemic. Validated questionnaires assessed physical activity, sitting time, and screen time. Linear regression quantified associations of COVID-19-related employment changes with physical activity, sitting time, and screen time, controlling for age, sex, race, BMI, smoking status, marital status, chronic conditions, household location, public health restrictions, and recalled physical activity, sitting time, and screen time prior to the COVID-19 pandemic.

**Results:** Compared to those whose employment remained unchanged, participants whose employment changed (either WFH or lost their job) due to COVID-19 reported higher sitting time (WFH: *g* = 0.153, 95% CI = 0.095–0.210; lost job: *g* = 0.212, 0.113–0.311) and screen time (WFH: *g* = 0.158, 0.104–0.212; lost job: *g* = 0.193, 0.102–0.285). There were no significant group differences for physical activity (WFH: *g* = −0.030, −0.101 to 0.042; lost job: *g*=-0.070, −0.178 to 0.037).

**Conclusion:** COVID-19 related employment changes were associated with greater sitting and screen time. As sedentary time is consistently negatively associated with current and future health and wellbeing, increased sedentary time due to employment changes is a public health concern.

## Introduction

Being regularly physically active has numerous physical and mental health benefits (1), and engaging in high sedentary time is negatively associated with many similar outcomes (2, 3). Previous population-based research has supported associations between employment status and type with activity behaviors. A cross-sectional analysis of data from 1,826 participants of the National Health and Nutrition Examination Survey (NHANES) showed that, among men, compared to not working, full-time employment in even predominately sedentary jobs, was positively associated with accelerometer-measured physical activity (4). Among men and women, those with active jobs showed greater weekday activity compared to those in sedentary jobs (4).

The novel coronavirus (SARS-COV-2) and associated disease (COVID-19) has significantly altered life, including employment, with many businesses/organizations temporarily or permanently closing and workers shifting to remote/virtual working environments. The National Bureau of Economic Research suggested that between February and May 2020 over 30% of the US labor force transitioned to working from home (WFH), and 10% of workers were laid off (5). Similarly, large changes in unemployment and working environments have been reported in Europe (6) and South East Asia (7). It is plausible that these significant alterations to employment status and working environment may alter activity behaviors. For example, some individuals WFH may find increased time and opportunities to engage in activity due to reduced commuting time, whereas others may significantly increase sedentary time, particularly screen-based time. The potential implications of COVID-19-related behavioral changes in activity behaviors have been widely discussed (8, 9); however, the extent to which COVID-19 related employment changes influence activity behaviors is unstudied.

Assessing how health risks of WFH are affected by its sudden, largescale uptake in the context of COVID-19 is essential to best preserve occupational health (10). Thus, this brief report explored whether COVID-19-related employment changes were associated with activity behaviors, including sitting time, screen time, and physical activity, in a sample of 2,303 previously employed US adult participants in the COVID-19 and Wellbeing (Cov-Well) Study.

## Methods

### Study Characteristics

This study uses cross-sectional data from The COVID-19 and Wellbeing (Cov-Well) Study. Details of the methodology employed by Cov-Well are fully described elsewhere (11). Briefly, Cov-Well is a survey including cross-sectional and longitudinal components which were approved as an exempt project by Iowa State University's Institutional Review Board (approval #:20-144-00). Convenience sampling using mass emails that included a link to an anonymous online survey to Iowa State University students, faculty, staff, and alumni, snowball sampling, and posts to social media pages were used to recruit potential participants. Participants considered for the current study completed the survey April 3rd–April 7th, 2020 and self-reported being employed prior to COVID-19 (*n* = 2,454). Participants with missing employment, activity, and covariate data were excluded (*n* = 54; 2.2%), as were those with implausible body mass index (BMI) and activity values (*n* = 97; 4.0%), leaving a final sample of 2,303.

### COVID-19-Related Employment Changes

The primary exposure was COVID-19-related employment change. Participants were asked “what is the impact of the recent events on your work life?” with possible answers “no change in work,” “working from home, when I was not before,” and “lost employment in relation to pandemic.”

### Physical Activity and Sedentary Behavior

The primary outcomes were current sitting time, screen time, and metabolic equivalent minutes (MET.mins) of physical activity. Participants reported the average daily time they spent sitting, engaged in moderate and vigorous physical activity (reported separately), and average daily screen-time. Time spent in physical activity was converted to MET.mins with moderate and vigorous intensity activity considered to be four and eight METs, respectively (12). This means that 1 min of moderate or vigorous intensity activity equated to expending four or eight times the amount of energy expended during a minute while at rest, respectively.

### Covariates

Covariates were age (10-year categories), sex (male, female, or transgender), race (white or other), BMI, smoking status (current smoker or not), marital status (married/in a relationship, widowed, separated/divorced, or never married), chronic conditions (summed into three categories: 0, 1, and ≥2), and COVID-19 public health restrictions (quarantined/required to quarantine/self-isolating, under a shelter-in-place/stay-at-home order, or social distancing). Depressive symptoms were assessed by the 21-item Beck Depression Inventory-II, excluding the suicidality question (range: 0–63). Participants recalled their average daily time spent sitting, engaged in moderate and vigorous physical activity, and average daily screen-time prior to the COVID-19 pandemic. Education was assessed but removed from primary analyses due to multicollinearity.

### Analyses

Data were analyzed in Stata version 14.2. Multivariable linear regression quantified adjusted associations [unstandardized betas (b) and standard errors (SEs)] of COVID-19-related employment changes with sitting time, screen time, and physical activity. Multicollinearity was determined as likely if two covariates had a correlation ≥0.8, the mean variance inflation factor (VIF) was ≥6, or the highest individual VIF was ≥10. Consequently, education was excluded from analyses. Robust standard errors, which are robust to heteroscedasticity, were used in the multivariable linear regression. Hedges' *g* effect sizes and associated 95% confidence intervals (95% CIs) were calculated such that more time in an activity was represented as a positive effect size.

## Results

**Figure 1:**
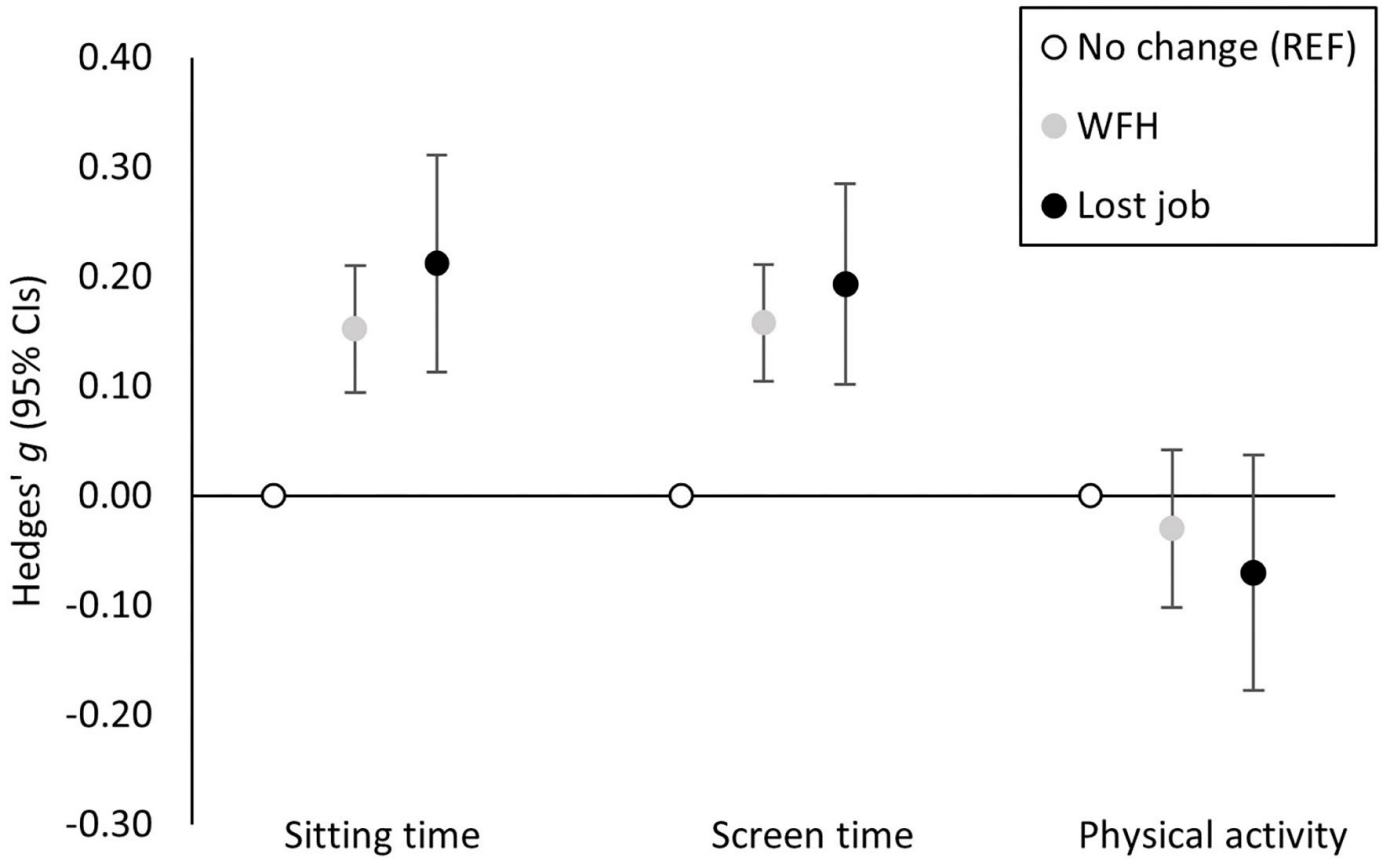
Hedges' g effect sizes and associated 95% confidence intervals (CIs) demonstrating the magnitude of the adjusted association between working from home (WFH) and job loss (compared to unchanged employment) and sitting time, screen time, and physical activity such that more time in a behavior is illustrated by a positive effect size. REF, reference category.

Participant (*n* = 2,303; 66% female) characteristics are presented in Table 1. Most (54%) were WFH when they were not before, 34% reported no change in employment, and 13% reported losing employment due to the pandemic. Mean ± SD activity times were: sitting time (533.0 ± 208.5 min/day), screen time (475.4 ± 224.8 min/day), and physical activity (471.9 ± 530.9 MET.min/day). Results from primary analyses for sitting time (F_(24, 2278)_ = 166.18, *p* < 0.001; R^2^ = 0.61), screen time (F_(24, 2278)_ = 289.20, *p* < 0.001; R^2^ = 0.71), and physical activity (F_(24, 2278)_ = 24.38, *p* < 0.001; R^2^ = 0.44) are presented in Figure 1 and full model outputs are reported in Supplementary Tables 1–3. Compared to those whose employment remained unchanged, participants switched to WFH or lost their job reported higher sitting time [WFH: b (SE) = 30.93 (5.98), *p* < 0.001; lost job: b (SE) = 44.24 (10.56), *p* < 0.001] and screen time [WFH: b (SE) = 34.15 (5.91), *p* < 0.001; lost job: b (SE) = 41.32 (9.97), *p* < 0.001]. There were no significant differences with physical activity [WFH: b (SE) = −15.41 (18.92), *p* = 0.415; lost job: b (SE) = −43.02 (33.63), *p* = 0.201].

**Table 1:** Participant characteristics by COVID-19-related changes in employment.

	**No change (*n* = 773)**	**WFH (*n* = 1,242)**	**Lost employment (*n* = 288)**
**Age (years)**			
18–24	103 (13.3)	183 (14.7)	139 (48.3)
25–34	112 (14.5)	321 (25.8)	22 (7.64)
34–44	99 (12.8)	280 (22.5)	25 (8.7)
45–54	116 (15.0)	206 (16.6)	34 (11.8)
55–64	157 (20.3)	183 (14.7)	36 (12.5)
65–74	126 (16.3)	57 (4.6)	25 (8.7)
≥75	60 (7.8)	12 (1.0)	7 (2.4)
Sex (female)	440 (56.9)	852 (68.6)	224 (77.8)
Race (white)	726 (93.9)	1143 (92.0)	258 (89.6)
BMI	27.3 ± 5.9	26.9 ± 6.0	25.9 ± 5.7
Smoker (yes)	28 (3.6)	20 (1.6)	14 (4.9)
Pre-COVID-19 screen time (mins)	313.2 ± 192.6	423.4 ± 204.0	279.4 ± 158.0
Pre-COVID-19 sitting time (mins)	411.2 ± 183.5	477.0 ± 171.5	364.8 ± 160.0
Pre-COVID-19 physical activity (MET.mins)	737.7 ± 730.8	514.3 ± 509.0	800.5 ± 660.1
**Marital status**			
Married/in a relationship	556 (71.9)	829 (66.7)	144 (50.0)
Widowed	18 (2.3)	15 (1.2)	3 (1.0)
Separated/divorced	56 (7.2)	67 (5.4)	11 (3.8)
Never married	143 (18.5)	331 (26.7)	130 (45.1)
**Education**			
Up to high school graduate	7 (0.9)	15 (1.2)	17 (5.9)
Up to college graduate	477 (61.7)	581 (46.8)	205 (71.2)
Graduate degree	289 (37.4)	646 (52.0)	66 (22.9)
Depressive symptoms	9.0 ± 8.7	10.3 ± 7.9	13.7 ± 9.9
**Chronic conditions**			
0	559 (72.3)	934 (75.2)	217 (75.3)
1	59 (7.6)	117 (9.4)	22 (7.6)
≥2	155 (20.1)	191 (15.4)	49 (17.0)
**Public health restrictions**			
Self-isolating/quarantining	111 (14.4)	212 (17.1)	73 (25.3)
Shelter in place	327 (42.3)	598 (48.15)	132 (45.8)
Social distancing	335 (43.3)	432 (34.78)	83 (28.8)

*Numbers are N (%) or mean ± standard deviation*.

*BMI, body mass index; MET.mins, metabolic equivalent minutes; mins, minutes; WFH, working from home*.

## Discussion

In this study of 2,303 US adults, switching to WFH and job loss due to the COVID-19 pandemic were associated with greater time spent sitting and viewing screens compared to those whose unemployment was unchanged. These associations are independent of self-reported time spent sitting, viewing screens, and engaging in physical activity prior to the pandemic. The magnitude of these associations was small-to-moderate and equated to ~31 and 33 min more sitting and screen time per day, respectively, among those WFH, and 44 and 40 min more sitting and screen time per day, respectively, among those who lost their jobs. Physical activity did not differ by COVID-19-employment changes. These findings, which were independent of prior sedentary time and physical activity, may have important implications for efforts to limit sedentary behaviors due to their established adverse effects on physical and mental health.

Switching to WFH was associated with more time each day spent in sedentary activities. Previous evidence has shown that office workers spend a higher percentage of their workday engaged in sedentary activity than their non-work hours (13). This somewhat supports the current findings, as greater sitting and screen time while WFH may be expected due to longer work hours, required screen-based meetings, and a lower ability to engage in activities outside of the home or office due to public health guidelines. Similarly, loss of job due to the COVID-19 pandemic was also associated with more time in sedentary behaviors, supporting previous evidence showing that full time employment was associated with lower leisure sedentary behavior (14). Increases in sedentary behavior are of major public health concern due to its known adverse effects on physical and mental health (2, 3). Employers have a duty to preserve the health of their employees, which may also be associated with productivity and days lost to illness in the workplace (15).

Previous evidence from NHANES showed that, among men, compared to not working, full-time employment was positively associated with accelerometer-measured physical activity (4); among men and women, those with active jobs showed greater weekday activity compared to those in sedentary jobs (4). However, these findings were not observed in the current study, with no differences in physical activity across employment-change categories observed. Substantial reductions in physical activity due to the COVID-19 pandemic have been reported, and it is likely that the pandemic environment may have a similar influence on physical activity across employment categories (11). Moreover, light intensity physical activity was not considered in the current study. Due to increased time spent sedentary and no differences in time spent in moderate and vigorous intensity physical activity, it is plausible that there have been reductions in light intensity physical activity which is a potential concern as it has been shown to benefit cardiometabolic health and may reduce overall mortality risk (16).

### Limitations

This study has several limitations. The cross-sectional design precludes inference of causality. The convenience sample is predominantly well-educated and white and so not entirely reflective of the total US population. Although well-validated questionnaires were used, physical activity and sedentary behaviors were self-reported and so potentially subject to misreporting (17).

### Implications

Changes in employment during the period of the COVID-19 pandemic resulted in higher self-reported sitting and screen time compared to those whose employment was unchanged. Future research should seek to replicate these findings in more diverse, nationally representative and international samples. Employers and public health bodies should consider health promotion through breaking up of sedentary time and increasing physical activity throughout the workday for those who have switched to WFH or lost their job due to the pandemic. As changing school environments result in more working parents having childcare duties throughout the workday, working from home is likely to continue and strategies that proactively address the associated potential increase in sedentary time are needed.

## Data Availability Statement

The raw data supporting the conclusions of this article will be made available by the authors, without undue reservation. Requests to access these data should be directed to Jacob Meyer, jdmeyer3@iastate.edu.

## Ethics Statement

The studies involving human participants were reviewed and approved by Iowa State University's Institutional Review Board. The patients/participants provided their written informed consent to participate in this study.

## Author Contributions

All authors study concept, design, and revision of the manuscript. CMcD, JM, and MH: analysis, interpretation of data, and drafting of the manuscript.

## Conflict of Interest

The authors declare that the research was conducted in the absence of any commercial or financial relationships that could be construed as a potential conflict of interest.

## References

[1] US Department of Health and Human Services Physical Activity Guidelines for Americans. 2nd ed. Washington, DC: US Department of Health and Human Services (2018).

[2] Celis-Morales CA Lyall DM Steell L Gray SR Iliodromiti S Anderson J. Associations of discretionary screen time with mortality, cardiovascular disease and cancer are attenuated by strength, fitness and physical activity: findings from the UK Biobank study. BMC Med. (2018) 16:77. 10.1186/s12916-018-1063-1 29792209 PMC5966877

[3] Stamatakis E Gale J Bauman A Ekelund U Hamer M Ding D. Sitting time, physical activity, and risk of mortality in adults. J Am Coll Cardiol. (2019) 73:2062–72. 10.1016/j.jacc.2019.02.031 31023430

[4] Van Domelen DR Koster A Caserotti P Brychta RJ Chen KY McClain JJ. Employment and physical activity in the US. Am J Prev Med. (2011) 41:136–45. 10.1016/j.amepre.2011.03.019 21767720 PMC5221416

[5] Brynjolfsson E Horton JJ Ozimek A Rock D Sharma G TuYe HY Covid-19 and Remote Work: An Early Look at Us Data. (2020). Available online at: https://www.nber.org/papers/w27344 (accessed July 27, 2020).

[6] European Foundation for the Improvement of Living and Working Conditions (Eurofound) Living, Working and COVID-19. (2020). Available online at: https://www.eurofound.europa.eu/publications/report/2020/living-working-and-covid-19 (accessed 29 September 2020).

[7] United Nations (UN) Policy Brief: The Impact of COVID-19 on South-East Asia. (2020). Available online at: https://unsdg.un.org/resources/policy-brief-impact-covid-19-south-east-asia (accessed 29 September 2020).

[8] Hall G Laddu DR Phillips SA Lavie CJ Arena R. A tale of two pandemics: how will COVID-19 and global trends in physical inactivity and sedentary behavior affect one another? Prog Cardiovasc Dis. (2020). 10.1016/j.pcad.2020.04.005. [Epub ahead of print]. 32277997 PMC7194897

[9] Ricci F Izzicupo P Moscucci F Sciomer S Maffei S Di Baldassarre A. Recommendations for physical inactivity and sedentary behavior during the coronavirus disease (COVID-19) pandemic. Front Public Health. (2020) 8:199. 10.3389/fpubh.2020.00199 32574294 PMC7235318

[10] Bouziri H Smith DR Descatha A Dab W Jean K. Working from home in the time of covid-19: how to best preserve occupational health? Occup Environ Med. (2020) 77:509–10. 10.1136/oemed-2020-106599 32354748 PMC7231547

[11] Meyer J McDowell C Lansing J Brower C Smith L Herring M Changes in Physical Activity and Sedentary Behaviour Due to the COVID-19 Outbreak and Associations With Mental Health in 3,052 US Adults. (2020). Available online at: https://www.cambridge.org/engage/coe/article-details/5eb2056d7a31fc00183d05db (accessed July 27, 2020).

[12] Craig CL Marshall AL Sjöström M Bauman AE Booth ML Ainsworth BE. International physical activity questionnaire: 12-country reliability and validity. Med Sci Sports Exerc. (2003) 35:1381–95. 10.1249/01.MSS.0000078924.61453.FB 12900694

[13] Prince SA Reed JL McFetridge C Tremblay MS Reid RD. Correlates of sedentary behaviour in adults: a systematic review. Obes Rev. (2017) 18:915–35. 10.1111/obr.12529 28524615

[14] Parry S Straker L. The contribution of office work to sedentary behaviour associated risk. BMC Public Health. (2013) 13:296. 10.1186/1471-2458-13-296 23557495 PMC3651291

[15] Aldana SG Pronk NP. Health promotion programs, modifiable health risks, and employee absenteeism. J Occup Environ Med. (2001) 43:36–46. 10.1097/00043764-200101000-00009 11201768

[16] Powell C Browne LD Carson BP Dowd KP Perry IJ Kearney PM. Use of compositional data analysis to show estimated changes in cardiometabolic health by reallocating time to light-intensity physical activity in older adults. Sports Med. (2020) 50:205–17. 10.1007/s40279-019-01153-2 31350674

[17] Dowd KP Szeklicki R Minetto MA Murphy MH Polito A Ghigo E. A systematic literature review of reviews on techniques for physical activity measurement in adults: a DEDIPAC study. Int J Behav Nutr Phys Act. (2018) 15:15. 10.1186/s12966-017-0636-2 29422051 PMC5806271

