# Supplementary material for: Working From Home and Job Loss Due to the COVID-19 Pandemic Are Associated With Greater Time in Sedentary Behaviors
Source: Front Public Health. 2020 Nov 5;8:597619. doi: 10.3389/fpubh.2020.597619 (PMC7674395; doi:10.3389/fpubh.2020.597619)
Supplement: Supplementary file 1 [file Table_1.DOCX]

| **Supplementary Table 1**. Associations (unstandardised betas (b) and associated standard errors (SEs)) of COVID-19-related employment changes with sitting time estimated by multivariable linear regression | | | |
| --- | --- | --- | --- |
|  | b | SE | P-value |
| Employment change |  |  |  |
| No change | **REF** |  |  |
| WFH | 30.93 | 5.98 | <0.001 |
| Lost employment | 44.25 | 10.56 | <0.001 |
| Age (years) |  |  |  |
| 18-24 | **REF** |  |  |
| 25-34 | -48.69 | 10.61 | <0.001 |
| 34-44 | -73.05 | 12.10 | <0.001 |
| 45-54 | -56.10 | 11.97 | <0.001 |
| 55-64 | -65.29 | 12.00 | <0.001 |
| 65-74 | -64.65 | 13.16 | <0.001 |
| ≥75 | -50.99 | 16.35 | 0.002 |
| Sex |  |  |  |
| Male | **REF** |  |  |
| Female | 0.86 | 6.24 | 0.890 |
| Other | -20.77 | 53.39 | 0.697 |
| Race (white) |  |  |  |
| White | **REF** |  |  |
| Other | 29.33 | 10.92 | 0.007 |
| BMI |  |  |  |
| Smoker (yes) |  |  |  |
| Yes | **REF** |  |  |
| No | 27.25 | 21.47 | 0.205 |
| Pre-COVID-19 screen time (mins) | 0.04 | 0.02 | 0.048 |
| Pre-COVID-19 sitting time (mins) | 0.83 | 0.02 | <0.001 |
| Pre-COVID-19 physical activity (MET.mins) | 0.01 | 0.01 | 0.008 |
| Marital status |  |  |  |
| Married/in a relationship | **REF** |  |  |
| Widowed | -14.49 | 22.26 | 0.515 |
| Separated/divorced | 25.62 | 10.97 | 0.020 |
| Never married | 21.11 | 8.56 | 0.014 |
| Depressive symptoms | 3.11 | 0.39 | <0.001 |
| Chronic conditions |  |  |  |
| 0 | **REF** |  |  |
| 1 | -10.40 | 9.44 | 0.271 |
| ≥2 | -4.34 | 8.19 | 0.596 |
| Public health restrictions |  |  |  |
| Self-isolating/quarantining | **REF** |  |  |
| Shelter in place | -21.93 | 8.50 | 0.010 |
| Social distancing | -36.00 | 8.78 | <0.001 |
| BMI=body mass index; MET.mins= metabolic equivalent minutes; mins=minutes; REF=reference category; WFH=working from home | | | |

| **Supplementary Table 2**. Associations (unstandardised betas (b) and associated standard errors (SEs)) of COVID-19-related employment changes with screen time estimated by multivariable linear regression | | | |
| --- | --- | --- | --- |
|  | b | SE | P-value |
| Employment change |  |  |  |
| No change | **REF** |  |  |
| WFH | 34.15 | 5.91 | <0.001 |
| Lost employment | 41.33 | 9.97 | <0.001 |
| Age (years) |  |  |  |
| 18-24 | **REF** |  |  |
| 25-34 | -54.08 | 10.35 | <0.001 |
| 34-44 | -74.70 | 11.38 | <0.001 |
| 45-54 | -71.40 | 11.55 | <0.001 |
| 55-64 | -70.87 | 11.92 | <0.001 |
| 65-74 | -78.25 | 12.97 | <0.001 |
| ≥75 | -87.31 | 15.13 | <0.001 |
| Sex |  |  |  |
| Male | **REF** |  |  |
| Female | -5.46 | 5.74 | 0.341 |
| Other | -55.03 | 44.92 | 0.221 |
| Race (white) |  |  |  |
| White | **REF** |  |  |
| Other | 20.17 | 9.91 | 0.042 |
| BMI |  |  |  |
| Smoker (yes) |  |  |  |
| Yes | **REF** |  |  |
| No | 12.92 | 18.90 | 0.494 |
| Pre-COVID-19 screen time (mins) | 0.85 | 0.02 | <0.001 |
| Pre-COVID-19 sitting time (mins) | 0.04 | 0.02 | 0.031 |
| Pre-COVID-19 physical activity (MET.mins) | 0.01 | <0.01 | 0.042 |
| Marital status |  |  |  |
| Married/in a relationship | **REF** |  |  |
| Widowed | 33.52 | 18.15 | 0.065 |
| Separated/divorced | 18.60 | 10.66 | 0.081 |
| Never married | 22.40 | 7.94 | 0.005 |
| Depressive symptoms | 3.12 | 0.37 | <0.001 |
| Chronic conditions |  |  |  |
| 0 | **REF** |  |  |
| 1 | -13.89 | 9.36 | 0.138 |
| ≥2 | 10.27 | 7.32 | 0.161 |
| Public health restrictions |  |  |  |
| Self-isolating/quarantining | **REF** |  |  |
| Shelter in place | -12.93 | 7.84 | 0.099 |
| Social distancing | -21.59 | 8.25 | 0.009 |
| BMI=body mass index; MET.mins= metabolic equivalent minutes; mins=minutes; REF=reference category; WFH=working from home | | | |

| **Supplementary Table 3**. Associations (unstandardised betas (b) and associated standard errors (SEs)) of COVID-19-related employment changes with physical activity time estimated by multivariable linear regression | | | |
| --- | --- | --- | --- |
|  | b | SE | P-value |
| Employment change |  |  |  |
| No change | **REF** |  |  |
| WFH | -15.41 | 18.92 | 0.415 |
| Lost employment | -43.02 | 33.63 | 0.201 |
| Age (years) |  |  |  |
| 18-24 | **REF** |  |  |
| 25-34 | 92.12 | 30.43 | 0.002 |
| 34-44 | 76.76 | 31.16 | 0.014 |
| 45-54 | 125.61 | 37.38 | 0.001 |
| 55-64 | 102.06 | 36.55 | 0.005 |
| 65-74 | 119.41 | 43.02 | 0.006 |
| ≥75 | 31.22 | 65.20 | 0.632 |
| Sex |  |  |  |
| Male | **REF** |  |  |
| Female | 29.88 | 18.75 | 0.111 |
| Other | 37.01 | 113.59 | 0.745 |
| Race (white) |  |  |  |
| White | **REF** |  |  |
| Other | -30.72 | 35.20 | 0.383 |
| BMI | -3.92 | 1.44 | 0.006 |
| Smoker (yes) |  |  |  |
| Yes | **REF** |  |  |
| No | 9.59 | 55.14 | 0.862 |
| Pre-COVID-19 screen time (mins) | -0.05 | 0.05 | 0.327 |
| Pre-COVID-19 sitting time (mins) | -0.19 | 0.05 | <0.001 |
| Pre-COVID-19 physical activity (MET.mins) | 0.53 | 0.03 | <0.001 |
| Marital status |  |  |  |
| Married/in a relationship | **REF** |  |  |
| Widowed | 29.09 | 81.59 | 0.722 |
| Separated/divorced | -33.16 | 38.88 | 0.394 |
| Never married | 20.62 | 23.89 | 0.388 |
| Depressive symptoms | -7.67 | 1.09 | <0.001 |
| Chronic conditions |  |  |  |
| 0 | **REF** |  |  |
| 1 | -7.34 | 32.00 | 0.819 |
| ≥2 | -12.99 | 24.18 | 0.591 |
| Public health restrictions |  |  |  |
| Self-isolating/quarantining | **REF** |  |  |
| Shelter in place | 72.18 | 25.06 | 0.004 |
| Social distancing | 62.55 | 25.50 | 0.014 |
| BMI=body mass index; MET.mins= metabolic equivalent minutes; mins=minutes; REF=reference category; WFH=working from home | | | |
